# Efficacy and Safety of Different Aerobic Exercise Intensities in Patients With Heart Failure With Reduced Ejection Fraction: Design of a Multicenter Randomized Controlled Trial (HF-EI Trial)

**DOI:** 10.3389/fcvm.2021.705972

**Published:** 2021-08-26

**Authors:** Ting Shen, Xiaoling Liu, Bo Zhuang, Qian Luo, Yishan Jin, Guanghe Li, Yumei Jiang, Dejie Li, Xianchuan Chen, Nuo Tang, Zhimin Xu, Lemin Wang, Liang Zheng, Yuqin Shen

**Affiliations:** ^1^Department of Rehabilitation, Tongji Hospital Affiliated to Tongji University, Tongji University School of Medicine, Shanghai, China; ^2^Department of Geriatrics, Yueyang Integrated Traditional Chinese Medicine and Western Medicine Hospital Affiliated to Shanghai University of Traditional Chinese Medicine, Shanghai, China; ^3^Department of Cardiology, Longhua Hospital Affiliated to Shanghai University of Traditional Chinese Medicine, Shanghai, China; ^4^Department of Cardiovascular Medicine, Xinhua Hospital Affiliated to Shanghai Jiao Tong University School of Medicine, Shanghai, China; ^5^Department of Cardiovascular Medicine, Research Center for Translational Medicine, Shanghai East Hospital, Tongji University School of Medicine, Shanghai, China

**Keywords:** exercise intensities, HFREF, randomized controlled trial, anaerobic threshold, exercise training

## Abstract

**Background:** Heart failure (HF) is one of the major causes of mortality worldwide, representing the terminal stage of several cardiovascular diseases. Exercise-based rehabilitation is a beneficial therapy for patients with chronic heart failure (CHF). However, there is a lack of specific guidance on clinical decision-making regarding optimal exercise intensity. It is necessary to optimize the clinical recommendations for HF exercises. We will evaluate the efficacy and safety of different aerobic exercise intensities in patients with heart failure with reduced ejection fraction (HFrEF): the HF-EI trial. This trial aims to assess the appropriate exercise intensity for patients with HFrEF.

**Methods:** After a baseline assessment to determine the safety of exercise, 180 patients will be randomly assigned to supervised high-intensity exercise training (ET) group, supervised moderate intensity training (MIT) group, and control group at a ratio of 1:1:1. Patients randomly receiving high intensity training (HIT) undergo supervised ET (3 times/week, 30 min) for aerobic endurance at 70% peak oxygen consumption (peak VO_2_) intensity for 12 weeks. The MIT patients will perform supervised aerobic ET (3 times/week, 35–42 min) at the anaerobic threshold (AT) intensity for 12 weeks. The control group will continue to maintain their daily activities and will not receive ET. During the baseline and follow-up period, physical examination, laboratory tests, cardiology diagnostic tests, cardiopulmonary exercise tests (CPET), 6-min walk distance (6MWD), scale scores, exercise steps, medications, and clinical events will be monitored. Throughout the research, sport bracelets and patient diaries will be used to monitor and record overall physical activity, training courses, and compliance.

**Discussion:** The HF-EI trial will evaluate the effects of different aerobic exercise intensities on peak VO_2_, quality of life (QoL), and clinical events among patients with HFrEF. The findings of this trial will provide a basis for formulating exercise prescriptions for patients with HFrEF.

**Clinical Trial Registration:**http://www.chictr.org.cn/, identifier: ChiCTR2000036381.

## Introduction

Chronic heart failure (CHF) is a severe and end-stage manifestation of several cardiovascular diseases. It is a syndrome with a high hospitalization rate, high disability rate, and high mortality rate, causing an economic and social burden on families (1). According to statistics, there are 64.3 million heart failure (HF) patients worldwide. In developed countries, the prevalence of adult HF is 1–2% (2). The China Hypertension Survey (CHS) showed that 1.3% of Chinese people aged ≥35 years, or an estimated 13.7 million individuals had HF (3). At present, there are many treatment option [s]s for HF, including general therapy, medication, and non-drug treatment (4). Despite active exploration of new medical and non-drug treatments for HF, the final effect is still unsatisfactory. The challenges facing HF intervention include optimizing HF treatment and reducing overall costs associated with long-term management.

Exercise training (ET) is a safe and effective treatment for HF. As shown in previous randomized controlled trials, it can significantly improve exercise capacity and quality of life (QoL) (5). Furthermore, ET is associated with reductions in mortality and hospitalization and recommended in the current guidelines on CHF management (6).

A massive amount of evidence suggests that ET is beneficial for patients with HF (7–16). A study by Belardinelli et al. in 2012 showed that exercise rehabilitation for up to 10 years could significantly reduce the readmission rate and the risk of cardiovascular death (17). A Cochrane review in 2019 found that exercise-based cardiac rehabilitation (CR) may have little effect on short-term all-cause mortality but may improve long-term all-cause mortality (>12 months follow up).CR probably reduces overall hospitalization rates in the short term (6).

However, data from the Exercise Training Meta-Analysis of Trials in Chronic Heart Failure (ExTraMATCH) II trial with at least 3 weeks of ET in HF showed that exercise-based CR had no significant effect on the risk of mortality and hospitalization in heart failure with reduced ejection fraction (HFrEF) (18).

Studies on the effects of exercise-based CR on the mortality and hospitalization rate of patients with HF show conflicting results, possibly attributed to differences in exercise pattern, exercise intensity, patient population selection, compliance, and follow-up time. All factors, especially exercise intensity, may affect the endpoint effect. Therefore, further research is needed to determine optimal exercise intensity to reduce the mortality and HF hospitalization rate.

Since the 1980s, numerous studies have demonstrated the safety and effectiveness of moderate intensity training (MIT) (19, 20). However, recent data suggest that high intensity training (HIT) may offer some advantages over MIT (21–32). Within a specific intensity range, exercise intensity is proportional to the effect. However, higher exercise intensity is associated with higher risk, and a higher need for adjusted to ensure safety and effectiveness in patients with HF. The mainstream sports rehabilitation model in developed countries of Europe and the United States is based on rehabilitation centers and ET under electrocardiogram (ECG) monitoring. A 12-week, three times a week treadmill exercise is used as a classic exercise prescription (33). The advantage is that it considers the efficacy and safety of ET, providing tremendous benefits to patients with HF in the short term. Keteyian et al. found that a moderate amount of exercise (3–7 metabolic equivalent hours per week) in patients with HF reduces the risk of clinical events, and within this range, an increase in the amount of exercise is associated with increased benefits (34). Swank et al. also obtained similar results. After exercise rehabilitation in patients with HF, increased peak oxygen consumption (peak VO_2_) was associated with improved prognosis (35).

According to the grading standards for exercise intensity proposed by the 2020 European Society of Cardiology (ESC) Guidelines on sports cardiology and exercise in patients with cardiovascular disease, the anaerobic threshold (AT) intensity is moderate, 70% PeakVO_2_ intensity is high intensity (36). We will design a three-arm randomized controlled experiment to explore the effectiveness and safety of moderate exercise intensity (AT intensity) in the treatment of HFrEF. The HIT (70% PeakVO_2_ intensity) group served as the active control group, and the daily activity group served as the placebo control group. Mainly compare MIT and HIT, or MIT and daily activities, and explore the dose-effect relationship of exercise intensity on changes in clinical outcomes through regression analysis. We assume that MIT has a greater impact in the main results than HIT and daily activities.

We will evaluate the efficacy and safety of different aerobic exercise intensities in patients with HFrEF: the HF-EI trial. This study is the first multicenter randomized controlled trial (RCT) on the effects of different aerobic exercise intensities in patients with HFrEF in China. By collecting data on exercise capacity and exercise rehabilitation, we will evaluate the clinical efficacy of exercise-based CR in Chinese patients with HFrEF. Finally, suitable exercise intensity will be recommended for the popularized HFrEF aerobic exercise program for Chinese patients with HFrEF.

The primary objective of the HF-EI trial is to investigate the effects of different exercise intensities on exercise capacity and quality of life in patients with HFrEF. As secondary objectives, the HF-EI trial will evaluate AT oxygen uptake, 6-min walk distance (6MWD), left ventricle ejection fraction (LVEF), N-terminal prohormone of brain natriuretic peptide (NT pro-BNP), the Patient Health Questionnaire-9 (PHQ-9), General Anxiety Disorder-7(GAD-7), all-cause mortality, HF hospitalization, major cardiovascular events, and adverse events.

## Methods/Design

### Design

**Figure 1:**
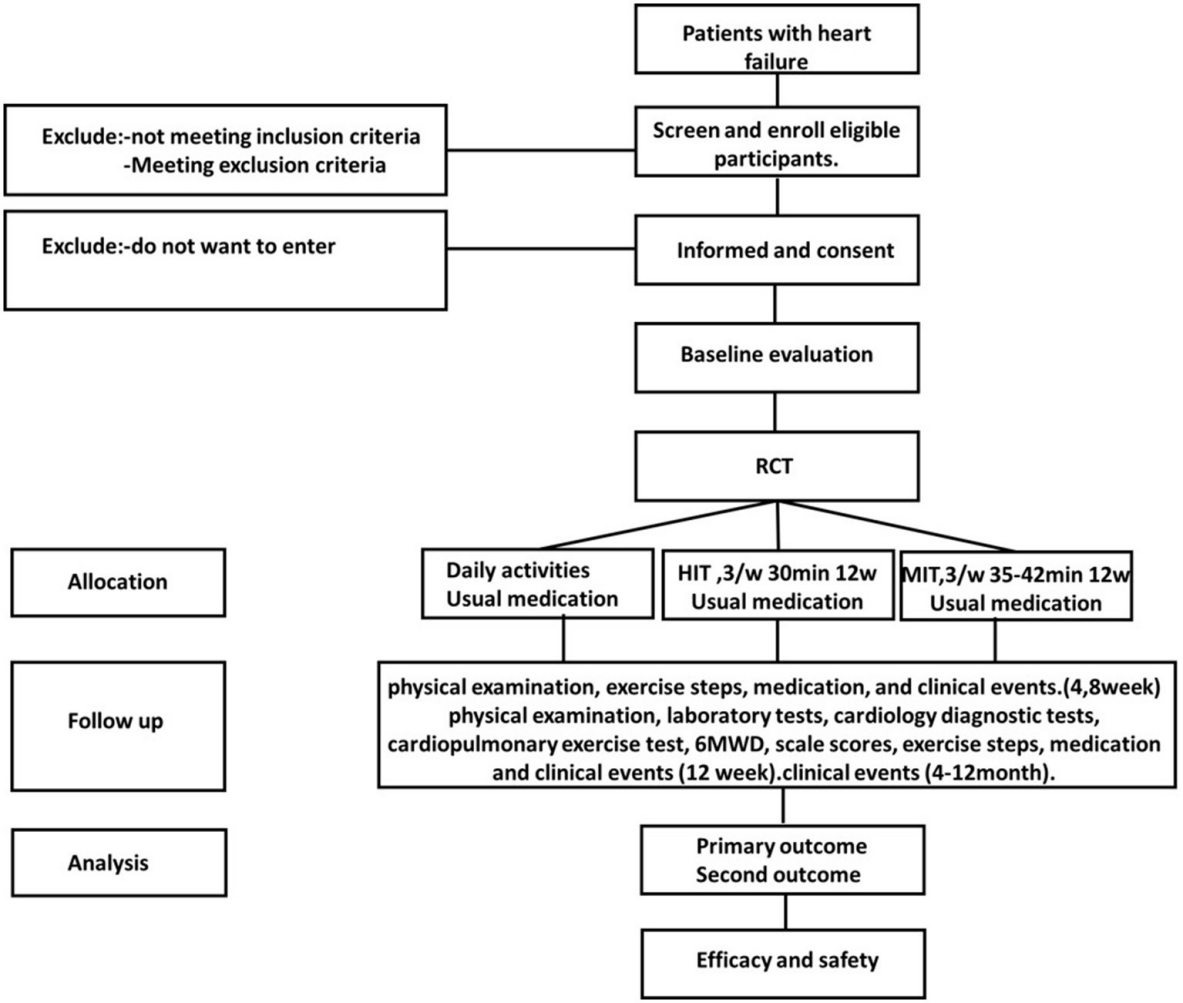
Flow chart of the study. RCT, randomised controlled trial; HIT, high intensity training; MIT, moderate intensity training.

**Figure 2:**
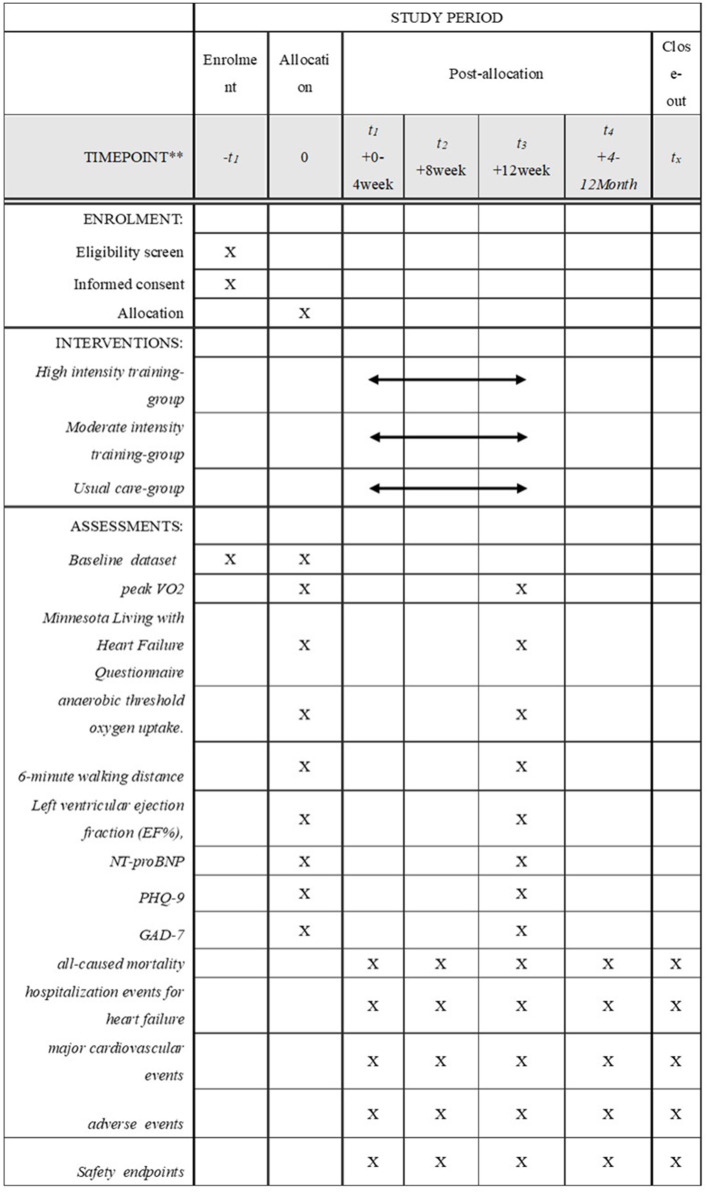
Standard protocol items. Peak VO_2_, peak oxygen consumption; NT-proBNP, N-terminal prohormone of brain natriuretic peptide; PHQ-9, Patient Health Questionnaire-9;GAD-7, General Anxiety Disorder-7.

This will be a multi-center, parallel, three-group RCT to evaluate the efficacy and safety of MIT for HFrEF. Eligible participants will be randomly assigned to the HIT intervention group receiving a 12-week supervised training sessions (70% PeakVO_2_ intensity) plus usual medications, MIT intervention group receiving a 12-week supervised training sessions (AT intensity) plus usual medications, or a control group receiving only the usual medication and maintain daily activity. This study has a 12-week intervention and a 12-month follow-up period. Figure 1 shows the study design flow chart. This protocol follows the Standard Protocol Items: Recommendations for Interventional Trials (SPIRIT) guidelines and fulfills the SPIRIT checklist (Supplemental File 1); A SPIRIT checklist is provided in Figure 2 (37).

### Setting and Participants

The HF-EI trial aims to recruit 180 stable HFrEF patients in three study sites (Tongji Hospital Affiliated to Tongji University, Yueyang Integrated Traditional Chinese Medicine and Western Medicine Hospital Affiliated to Shanghai University of Traditional Chinese Medicine, and Longhua Hospital Affiliated to Shanghai University of Traditional Chinese Medicine) in China and apply standard therapy in accordance with the ESC HF guidelines. We have predefined a set of inclusion and exclusion criteria (Table 1) to ensure the feasibility and safety of ET. The main method of recruiting patients is to screen the patients on-site when visiting a doctor.

**Table 1:** HF-EI trial inclusion and exclusion criteria.

**Inclusion criteria**
1. Aged 18 to 75 years old
2. BMI 18–30 kg/m^2^
3. LVEF <0.4
4. NYHA I-III.
5. Clinical symptoms are stable for at least 2 weeks
6. The dose of angiotensin-converting enzyme inhibitor/angiotensin receptor antagonist/angiotensin receptor enkephinase inhibitor, beta-blocker, aldosterone inhibitor and other drugs was stabilized for at least 2 weeks.
7. Written informed consent.
**Exclusion criteria**
1. Have a history of heart implant device in the last 6 weeks, or have a heart implant device plan or heart transplant plan within the next 12 months
2. Acute coronary syndrome within 6 weeks
3. Severe valvular heart disease, congenital heart disease, severe hypertrophic obstructive cardiomyopathy, acute myocarditis/pericarditis, intracardiac thrombosis, primary pulmonary hypertension, perinatal cardiomyopathy, thyroid heart disease;
4. Implanted pacemaker with no frequency response function, or set the discharge threshold of ICD lower than the heart rate during exercise.
5. Contraindications to exercise training: uncontrolled hypertension (systolic blood pressure >200 mmHg and/or diastolic blood pressure >110 mmHg); Severe arrhythmias such as ventricular tachycardia, frequent multi-source premature ventricular beats, high atrioventricular block and significant QT interval prolongation occurred during exercise test.
6. Progressive dyspnea or loss of exercise endurance in the last 3–5 days at rest;
7. Myocardial ischemia under low power exercise load (<2MET, or <50W);
8. Uncontrolled diabetes;.
9. Recent embolism;
10. Thrombophlebitis;
11. New atrial fibrillation or atrial flutter, etc., and other conditions not suitable for sports training;
12. Severe liver and kidney dysfunction, liver function more than three times the normal value, eGFR <30 mL/min/1.73 m2;
13. Currently suffering from severe mental illness, severe respiratory disorders, severe hematological diseases, severe nervous and neuromuscular diseases, severe metabolic and endocrine diseases, malignant neoplasms, severe autoimmune diseases, complicated with systemic infections and other acute systemic diseases.
14. Other clinical studies have been included to influence the results of this study;
15. Women who are pregnant or preparing to become pregnant, and women who are breastfeeding;
16. The researchers determined that the patients were not suitable for the study.

*BMI, body mass index; LVEF, left ventricular ejection fraction; NYHA, New York Heart Association; ICD, implantable cardioverter-defibrillator; MET metabolic equivalent; eGFR, estimated glomerular filtration rate*.

### Randomization and Allocation

We will use a block- randomization, stratified in groups for center to assign participants to the high- intensity exercise group, moderate- intensity exercise group and the control group in a 1:1:1 ratio. For the hierarchical random assignment operation, the statistical unit responsible adopted the SAS version 9.2 statistical software (procedure 'PROC PLAN') to complete program writing and randomization operations. The random distribution results will be released through the network of a central random distribution system. The results of random allocation will be managed by a person designated by the research group, and the results will be executed independently by clinicians. Due to the interventional study design, blinding is impossible for patients and clinical operators. However, the examiners, researchers collecting data on outcome indicators, data managers, and statisticians will be blinded.

### Intervention and Control

The training program of intervention groups and the control group is shown in Figure 2. The HF-EI trial aims to ensure that patients receive appropriate and stable guide-based care before entering the trial. Throughout the trial, the three groups of patients will receive the usual medications, monthly follow-ups and the same number of visits. This study has a 12-week intervention and a 12-month follow-up period. All interventions and follow-ups will be completed by doctors (obtained a licensed physician qualification) in the Heart Rehabilitation Center.

### Control Group

Patients in the control group will continue to maintain their daily activities (daily activities monitor the number of exercise steps through a sport bracelet) and will not provide additional ET.

### Intervention Group

Patients randomized to receive HIT or MIT training receive 36 supervised training sessions, thrice weekly, in addition to usual care for 12 consecutive weeks of aerobic ET. Patients will be regularly encouraged to exercise on other days. The duration of each exercise session in the high intensity training group is 30 min, and the duration of each exercise session in the moderate intensity training group is 35–42 min. It is estimated that the high intensity training group and the moderate intensity training group have similar energy expenditure (38, 39). Table 2 summarizes the training program for the HF-EI trial. Participants who have serious adverse clinical events during the intervention will immediately stop the intervention measures and withdraw from this clinical study.

**Table 2:** Training program.

**Group**	**Intervention**			
	**Frequency**	**Duration**	**Intensity**	**Form**
High intensity training group	3 times per week	30 min	70% peak VO_2_	Centre-based rehabilitation: treadmill. Home-based cardiac rehabilitation: walk.
Moderate intensity training group	3 times per week	35–42 min	AT	Centre-based rehabilitation: treadmill. Home-based cardiac rehabilitation: walk.
Control group	Daily activities.

*peak VO_2_, peak oxygen consumption; AT, anaerobic threshold*.

### Adherence

Adherence refers to the degree to which trial participants comply with the intervention plan. Patients need to complete at least 66% of possible supervised training courses before consideration for treatment. The HF-EI trial uses several methods to promote compliance. First, researchers and trainers should regularly encourage patients randomized to ET to participate in supervised training courses. Second, a diary is used to track patient attendance and training to monitor patient compliance throughout the trial.

### Outcome Measures

The primary outcome for the HF-EI trial is the change in peak VO_2_ and Minnesota Living with Heart Failure Questionnaire scores (MLHFQ)after 12 weeks. We choose the common primary outcomes in order to obtain the comprehensive effect of different exercise intensities on HFrEF treatment. Aerobic exercise has a well-known positive effect on patients with HFrEF in exercise capacity and quality of life, and peakVO_2_ is an important indicator for evaluating the prognosis of HF (22, 40).

Secondary outcomes included, AT oxygen uptake, 6MWD, LVEF, NT pro-BNP, PHQ-9, GAD-7, all-cause- mortality, hospitalization events for HF, major cardiovascular events, and adverse events.

The test indicators require patients to be measured at the hospital rehabilitation center. The test details are as follows:

#### Cardiopulmonary Exercise Testing(CPET)

According to the recommendations of international guidelines, CPET will be performed before randomization to determine exercise safety in patients. We will evaluate patient exercise endurance using CPET peak power, exercise duration, peak oxygen uptake, and AT. The core laboratory trains and certifies all researchers performing CPET before recruiting personnel.

#### 6-min Walking Test (6MWT)

Perform 6MWT according to the guidelines of the American Thoracic Society (41). In the room, follow a long and straight corridor and instruct patients to walk back and forth at their chosen walking speed. The corridor is 30 m long, with a mark every 3 m, and turning points are marked with cones. During the test, all patients are guided by a unified standard record.

#### Echocardiography

Echocardiographic images and loops will be digitally recorded and stored on-site for analysis in an assessor-blinded reference echocardiography core lab, using color Doppler ultrasound diagnostic equipment, probe frequency 2.5–3.5HZ. During the examination, the patient assumes the left lateral decubitus position to obtain, the left ventricular long-axis view and the left ventricular short-axis view. The sampling lines are collected at the papillary muscle levels, the level of the mitral valve, and the aortic root to acquire two-dimensional images and an M-shaped spectrum. Echocardiography is repeated 12 weeks after randomization.

#### Laboratory Measurements and Biobanking

After resting for at least 5 min, blood samples will be collected under standardized conditions. According to standard operating procedures, all samples will be centrifuged immediately, aliquoted, and stored at −80 °C. Blood tests will be performed by a locally accredited laboratory.

#### Psychometric Analysis

A validated standard questionnaire will be used to assess the quality of life at baseline with repeat assessments within 12 weeks of patient randomization.

### Data Collections

The HF-EI research data collection process is shown in Figure 2.

#### Baseline Data

We will monitor the demographic data, medical history, physical status, laboratory tests (NT pro-BNP), cardiology diagnostic tests (LVEF), cardiopulmonary exercise tests (peak VO_2_, AT oxygen uptake), 6MWD, scale score(MLHFQ, PHQ-9, and GAD-7)and medications during the baseline period. Baseline data will be collected through outpatient consultation and medical record review.

#### Outcome Assessments

Physical examination, exercise steps, medication use, and clinical events (all-cause-mortality, hospitalization events for HF, major cardiovascular events, and adverse events) will receive outpatient follow-up in the 4th and 8th weeks after random assignment. At 12 weeks after randomization, physical examination, laboratory tests, cardiology diagnostic tests, cardiopulmonary exercise test, 6MWD, scale scores, exercise steps, medication, and clinical events will receive outpatient follow-up visits. 4–12 months after randomization, clinical events will be followed up by telephone every month. Both the baseline survey and follow-up will be conducted by two physicians.

### Statistical Methods

The calculation of the sample size will be based on the primary outcome of the RCT. The sample size calculation was performed using PASS V11.0 (NCSS Company of the United States) with the following calculation formula:

$$\begin{array}{l} n=\Psi^{2}\left[\sum_{i=1}^{k}s_{i}^{2}/k\right]/\left[\sum_{i=1}^{k}{\left({\bar{X}}_{i}-\bar{X}\right)}^{2}/\left(k-1\right)\right]\left(k\;=\;3\right) \end{array}$$

The parameters are set to double-sided α = 0.05, β = 0.1, Power = 1–β = 90%, the average difference in peak VO_2_ of the three groups were 0, 2, 4(ml/kg/min) and the average difference in MLHFQ of the three groups were 2, 12, 10. The corresponding standard difference values of peakVO_2_ were 6, 4, 6 (ml/kg/min) and MLHFQ were 5, 18, 16.

The parameter estimation is based on the results of previous researches (22, 42, 43).

A total of 180 cases were required for the three groups, 60 patients in each group, considering the 20% loss to follow-up rate and stratification factors. Because each of the primary endpoints can be individually reflected in clinical significance. Therefore, if one of the two primary endpoints of the intervention group improves significantly, the study is successful.

Blinded statistical analysis will be performed by qualified statisticians using PASW Statistics 18.0 (IBM SPSS Inc., Armonk, New York, USA). The measurement data will be expressed as mean ± standard deviation (x ± s), measurement data by *t* test, and count data by chi-square test. The clinical events of the treatment groups will be statistically compared, according to the principle of intention-to-treat. The Kaplan–Meier method will be used to calculate the cumulative event rate. The event time of all patients will be measured starting from the randomization time (time zero). We will collect all available information on clinical events until the last patient contact, including patients who withdraw consent or those lost to follow-up. Relative risk (RR) will be calculated using the Cox proportional hazard model. If a sufficient number of events occur, the Kaplan–Meier curve will be used for stratified analysis of the exercise group, and the log-rank test will be used to test the difference in survival. The significance level will be set at *p* < 0.05.

### Trial Management and Quality Control

The management structure includes a trial management team, a data monitoring committee, and a trial steering committee.

The trial management team (including two physicians and three investigators) is responsible for the implementation of the trial and will meet weekly to discuss the progress of the trial. The data monitoring committee is composed of three clinicians and two biostatisticians, and independently evaluates the safety, scientific validity and completeness of clinical trials. The data monitoring committee will convene a meeting before the start of patient recruitment and once a month after the patient begins to intervene. The person in charge of the center monitors the intermediate results during the clinical trial. Once serious side effects occur, the trial should be stopped immediately. If there are significant differences in the efficacy of the three groups (HIT group, MIT group, and control group), the trial will be considered to be terminated. Responsibility of the test steering committee is to approve the main research plans and revisions, supervise the test, guide the test, and solve the problems of the test management team. The committee will be composed of four experts and will meet at least once every 6 months.

Before recruitment, the entire research team needs to participate in a training seminar. The data collected in this trial will include questionnaire information, medical chart review and test results. Professionals double-enter the data and store the data confidentially in the electronic database. The person in charge of the sub-center has the right to access the data set of the sub-center, and the person in charge of the project has the right to access the final data set of all the centers. Researchers and sponsors will inform participants and other relevant personnel of the results of the trial by publishing articles. The data quality will be regularly checked by the research assistant and supervised by the supervisor.

### Trial Organization

Before enrolling patients, the protocol will be approved by the relevant institutional review boards, research ethics boards, and ethics committees of all the participating centers and the coordination center.

The clinical trial will be conducted in accordance with local laws. The study has been approved by Regional Committees for Medical Research Ethics of all participating centers (2020-KYSB-177). The study is registered at http://www.chictr.org.cn/ and the registration number is ChiCTR2000036381. The trial started in December 2020 and is currently recruiting.

## Discussion

The exercise-based CR is recommended in the guidelines for HFrEF but is still in infancy in China, where exercise-based CR services are rare in most regions, and the most suitable intensity ET for HFrEF patients remains unknown. Compared with global HFrEF exercise rehabilitation, HFrEF exercise rehabilitation in China started late and is still developing. Affected by factors, such as Chinese health and medical conditions and patients' exercise preferences, the overall level of CR in HFrEF still has some gaps compared with foreign countries. However, CR is a healthy behavior intervention affected by the social environment, cognition, and humanities. Simply introducing foreign experience cannot solve China's practical problems. The guidelines that are widely accepted abroad are referenced in China, but they need to be used in conjunction with China's actual adjustments. In this case, China currently lacks reference standards and effectiveness data on exercise intensity for HFrEF rehabilitation. The results of multi-center, randomized controlled registration studies from China are urgently needed to provide high-quality evidence and data from China.

To our knowledge, this is the first multicenter randomized controlled trial protocol to explore the appropriate exercise intensity for patients with HFrEF in China. The HF-EI trial will assess the impact of aerobic exercise intensity on the peak VO_2_ and QoL in patients with HFrEF. It also provides a rationale for improving functional capacity and cardiovascular prognosis in patients with HFrEF. We hope that this research fills the gap in the literature and offers high-quality evidence on the recommendations for the treatment of HFrEF.

AT relates to the point when the exercise load increases to a certain amount and the tissue demand for oxygen exceeds the amount that the circulation can provide, and the tissues undergo anaerobic metabolism to provide more energy. The critical point from aerobic metabolism to anaerobic metabolism is called AT, expressed as the threshold oxygen consumption of anaerobic metabolism (VO_2_ AT), equivalent to 50–60% of peak oxygen consumption, and directly detected by CPET (44). The advantages of using AT as the basis for formulating exercise prescriptions are sub-maximal exercise intensity, which can prevent the continuous increase of lactic acid levels, avoid hyperventilation and shortness of breath, avoid metabolic alkalosis, reduce the occurrence of overload on the heart and arrhythmia, and ensure high safety. Aerobic exercise with the intensity of the AT has protective effects on CHF, confirmed by the literature (45). For patients with HFrEF, exercise intensity is a range, not a point; AT belongs to sub-maximum medium intensity. Although 70% of peakVO_2_ is high- intensity, it is not ultra-high intensity. This study will explore the effects of these two different intensities.

AT intensity has not been widely accepted internationally. This study compares the effectiveness and safety of AT intensity and high intensity in patients with HF, and aims to reflect the superiority of AT intensity exercise. It has reference value for other colleagues in the world.

Limitations of the study should be recognized as follows: lost to follow-up, influencing factors including patient age, difficulties attending appointments, concurrent illnesses, and the nature of the intervention that made blinding impossible. We made every effort to ensure that evaluators, laboratory technicians and statisticians remain unaware of the treatment allocation.

Altogether, the results of the HF-EI trial may provide evidence on the effective delivery of a contextually adapted exercise-based CR program in China. At the same time, it provides evidence of AT exercise intensity for the world's CR field.

## Conclusions

The HF-EI trial will assess the effects of different aerobic exercise intensities on peak VO_2_ and QoL in patients with HFrEF. It will provide a rationale for improving functional capacity and cardiovascular outcomes in patients with HFrEF.

## Ethics Statement

The studies involving human participants were reviewed and approved by Shanghai Tongji Hospital Ethics Committee. The patients/participants provided their written informed consent to participate in this study.

## Author Contributions

TS and XL prepared the manuscript and all the authors participated in the design of clinical and related research. All authors gave final approval and agreed to be accountable for the integrity and accuracy of all aspects of the work.

## Conflict of Interest

The authors declare that the research was conducted in the absence of any commercial or financial relationships that could be construed as a potential conflict of interest.

## Publisher's Note

All claims expressed in this article are solely those of the authors and do not necessarily represent those of their affiliated organizations, or those of the publisher, the editors and the reviewers. Any product that may be evaluated in this article, or claim that may be made by its manufacturer, is not guaranteed or endorsed by the publisher.

## Abbreviations

6MWD: 6-min walk distance
6MWT: 6-min walking test
AT: anaerobic threshold
BMI: body mass index
CHF: chronic heart failure
CHS: China hypertension survey
CPET: cardiopulmonary exercise testing
CR: cardiac rehabilitation
ECG: electrocardiogram
eGFR: estimated glomerular filtration rate
ESC: European Society of Cardiology
ET: Exercise training
ExTraMATCH: Exercise Training Meta-Analysis of Trials in Chronic Heart Failure
GAD-7: General Anxiety Disorder-7
HF: heart failure
HF-EI trial: the efficacy and safety of different aerobic exercise intensities in patients with heart failure with reduced ejection fraction
HFrEF: heart failure with reduced ejection fraction
HIT: high intensity training
ICD: implantable cardioverter-defibrillator
LVEF: left ventricle ejection fraction
MET: metabolic equivalent
MIT: moderate intensity training
MLHFQ: Minnesota Living with Heart Failure Questionnaire scores
NT pro-BNP: N-terminal prohormone of brain natriuretic peptide
NYHA: New York Heart Association
Peak VO_2_: peak oxygen consumption
PHQ-9: the Patient Health Questionnaire-9
QoL: quality of life
RCT: randomized controlled trial
RR: relative risk
SPIRIT: Standard Protocol Items: Recommendations for Interventional Trials.
